# Monoclonal Antibodies and Small-Molecule Therapies for Lichen Planus: Targeted Immunomodulation and Emerging Evidence

**DOI:** 10.3390/antib14030079

**Published:** 2025-09-17

**Authors:** Francois Rosset, Nadia Sciamarrelli, Luca Mastorino, Valentina Pala, Sara Boskovic, Eleonora Bongiovanni, Orsola Crespi, Yingying Liao, Simone Ribero, Pietro Quaglino

**Affiliations:** School of Dermatology and Venereology, Department of Medical Sciences, University of Turin, 10126 Torino, Italy

**Keywords:** lichen planus, biologic therapy, monoclonal antibodies, JAK inhibitors, targeted immunotherapy, IL-17, TNF-alpha, immune modulation, precision medicine, autoimmune skin disease

## Abstract

**Background/Objectives:** Lichen planus (LP) is a chronic inflammatory disease of autoimmune origin, affecting the skin and mucous membranes. While corticosteroids and immunosuppressants are traditionally used, many cases remain refractory or intolerant to standard therapies. Recent advances in immunopathogenesis have led to the exploration of targeted therapies, including biologic agents and small-molecule inhibitors. **Methods:** This review synthesizes current evidence from case reports, case series, and observational studies on the use of monoclonal antibodies (anti-TNF-α, anti-IL-17, anti-IL-23, anti-IL-6) and JAK inhibitors in LP. A structured literature search was conducted across PubMed, Scopus, and Web of Science, focusing on studies published between 2010 and 2025. Data on mechanisms, clinical efficacy, safety, and research limitations were extracted and summarized. **Results:** Promising therapeutic responses were reported for IL-17 inhibitors (secukinumab, ixekizumab) and JAK inhibitors (tofacitinib, baricitinib) in mucosal and recalcitrant LP. Anti-TNF agents showed variable efficacy, while emerging targets such as BTK and IFN-γ are under investigation. Adverse events were generally mild to moderate, but long-term safety data are lacking. The absence of randomized controlled trials and standardized outcome measures limits generalizability. **Conclusions:** Biologic and small-molecule therapies represent a potential paradigm shift in the treatment of LP, offering targeted immunomodulation with promising efficacy in refractory cases. Further collaborative research, including randomized studies and biomarker-driven approaches, is urgently needed to validate these treatments and establish personalized care strategies.

## 1. Introduction

Lichen planus (LP) is a chronic inflammatory disease affecting the skin and mucous membranes, characterized histologically by a dense band-like lymphocytic infiltrate at the dermo-epidermal junction and clinically by pruritic, violaceous, polygonal papules [1]. The condition encompasses a heterogeneous group of subtypes including cutaneous LP, oral lichen planus (OLP), genital LP, nail LP, and lichen planopilaris (scalp involvement), each with distinct clinical manifestations and variable prognoses [2,3].

The epidemiology of LP remains incompletely understood, with a global prevalence estimated at approximately 1–2% in the general population [4]. Oral lichen planus is particularly common, representing one of the most frequent chronic inflammatory conditions of the oral mucosa. LP affects both sexes, though oral forms appear more prevalent in middle-aged women [2]. While cutaneous LP may resolve spontaneously within one to two years, mucosal involvement often persists and may be recalcitrant to therapy, significantly impairing quality of life due to pain, erosions, and functional limitations [2,5].

The pathogenesis of LP is complex and not yet fully elucidated, but increasing evidence supports an autoimmune-mediated mechanism [6]. Cytotoxic CD8+ T lymphocytes are considered key effectors, targeting basal keratinocytes perceived as antigenically altered, possibly due to viral infections, drug exposure, or autoantigens [7]. The interface dermatitis pattern seen histologically reflects this T-cell mediated cytotoxicity. Several pro-inflammatory cytokines, including interferon-γ [8,9], tumor necrosis factor-alpha (TNF-α), interleukin-17 (IL-17) [10], and interleukin-23 (IL-23), play roles in disease perpetuation. Moreover, intracellular signaling pathways such as Janus kinase/signal transducer and activator of transcription (JAK/STAT) are increasingly recognized as central drivers of the immune dysregulation observed in LP [7]. These molecular insights have paved the way for targeted therapies aimed at modulating specific immune pathways [6,7].

Despite the availability of topical [11] and systemic corticosteroids [12], immunosuppressants such as cyclosporine, methotrexate, and azathioprine, and topical calcineurin inhibitors, treatment of LP remains challenging [5,13]. Many patients experience relapsing disease or inadequate response, and prolonged use of conventional therapies may lead to adverse effects or contraindications, particularly in chronic mucosal involvement [5,14]. Moreover, oral and genital LP can be particularly refractory, and concerns persist regarding the risk of malignant transformation, especially in the erosive subtype of OLP [15].

The growing understanding of the immunopathogenesis of LP has fostered interest in novel therapeutic options that provide targeted immunomodulation with potentially improved efficacy and safety profiles [16]. In particular, biologic agents (such as monoclonal antibodies directed against IL-17, TNF-α, or IL-23) and small-molecule inhibitors of JAK pathways have shown promising results in case reports, small cohorts, and early-phase clinical studies [14]. However, the evidence remains fragmented, and treatment algorithms are still evolving [13].

This review aims to provide a comprehensive overview of the current state of biologic and small-molecule therapies for lichen planus. We focus on their mechanisms of action, clinical efficacy, safety data, and potential role in future treatment strategies. Additionally, we highlight the limitations of existing studies, propose research priorities, and discuss the potential of precision immunotherapy in the management of this challenging condition.

## 2. Materials and Methods

This narrative review was conducted to synthesize the available literature on biologic therapies and small-molecule inhibitors in the treatment of lichen planus (LP), with a focus on their immunological targets, clinical efficacy, and safety profiles. A systematic literature search was performed across three major biomedical databases—PubMed, Scopus, and Web of Science—to identify relevant peer-reviewed articles published between January 2010 and June 2025, reflecting the period during which most targeted therapies and immunomodulatory agents have emerged in dermatology and immunology. The database search yielded 390 records in total (PubMed, n = 182; Scopus, n = 121; Web of Science, n = 87). After de-duplication (n = 142), 248 unique records remained for screening.

The search included combinations of keywords and Medical Subject Headings (MeSH) such as: “lichen planus”, “oral lichen planus”, “cutaneous lichen planus”, “genital lichen planus”, “biologic therapy”, “monoclonal antibodies”, “anti-TNF”, “anti-IL-17”, “anti-IL-23”, “JAK inhibitors”, “small molecules”, “targeted therapy”, “autoimmune skin diseases”, and “immunomodulation”. Boolean operators (AND, OR) were used to refine and combine terms. Filters were applied to include articles published in English and human studies only.

Studies were included if they reported on the use of biologic agents or small-molecule therapies for any form of LP in human subjects. Eligible study types included randomized controlled trials (RCTs), cohort studies, case series, and case reports. Pre-clinical studies (in vitro or animal models) were included only when relevant to mechanism of action or translational relevance. Editorials, opinion pieces, and narrative reviews were excluded unless they contributed significant insights into emerging therapies or mechanistic pathways. Duplicate records were removed manually.

Title/abstract screening was conducted on 248 records, of which 174 were excluded. Seventy-four full-text articles were assessed for eligibility; 66 were excluded with reasons (not biologics/JAK/small-molecule focus, n = 28; non-interventional/mechanistic only, n = 18; insufficient outcome data, n = 12; non-English/erratum/other, n = 8). Eight studies met inclusion criteria for the therapeutic evidence synthesis summarized in Table 1 (5 primary interventional/safety studies and 3 systematic reviews). A qualitative synthesis was performed, with data extracted on study design, number of patients, LP subtype, treatment administered, outcomes measured, duration of follow-up, and reported adverse events. When available, findings were stratified by type of biologic agent or small molecule, and efficacy data were contextualized according to disease severity and subtype.

The review was conducted following principles aligned with PRISMA-ScR guidelines [17]; no formal meta-analysis was performed due to heterogeneity and the predominance of non-randomized studies.

During manuscript preparation, we used GPT-5 Thinking (ChatGPT, OpenAI) solely to assist with language/grammar checking and to help plan the review’s outline; all outputs were verified and edited by the authors, who assume full responsibility for the text.

**Table 1:** Evidence synthesis of therapeutic studies cited in the manuscript, organized by study design, lichen planus subtype, sample size, intervention, outcomes, and level of evidence according to OCEBM 2011 criteria.

Citation	Study Design	LP Subtype/Population	Sample Size	Intervention(s) vs. Comparator(s)	Primary Endpoint(s)	Level of Evidence
Lodi, 2020 (Cochrane) [12]	Systematic review & meta-analysis of RCTs	Adults with symptomatic oral lichen planus (OLP)	35 RCTs; 1474 participants	Topical corticosteroids (various) vs. placebo or active comparators; some trials TAC vs. clobetasol, TAC vs. triamcinolone, etc.	Pain reduction; clinical resolution; adverse effects	1a
Serafini, 2023 (IJERPH) [11]	Systematic review of RCTs (no meta-analysis)	Adults with symptomatic OLP	15 RCTs (total N varies)	Topical corticosteroids; calcineurin inhibitors; phytomedicines; PDT/LLLT; ozone; cryotherapy	Pain reduction; clinical resolution; adverse effects	1a
Vinay, 2024 (JAMA Dermatology) [18]	Randomized, double-blind, placebo-controlled RCT	Adults with symptomatic OLP, single center (India)	64 randomized	Oral acitretin (25–35 mg/day) + topical triamcinolone 0.1% vs. topical triamcinolone + oral placebo	Proportion achieving ODSS-75 at week 28 (and week 36)	1b
Passeron, 2024 (Br J Dermatol)—PRELUDE [19]	Randomized, double-blind, placebo-controlled phase II “basket” RCT	Adults with refractory CLP, MLP or LPP	111 randomized (37 per cohort)	Secukinumab 300 mg q4w × 32 wks vs. placebo × 16 wks (then secukinumab q2w)	IGA ≤ 2 at week 16 (by cohort)	1b
Hwang, 2025 (J Clin Invest) [20]	Phase II, single-arm, open-label	Adults with cutaneous LP (CLP)	12	Baricitinib 2 mg daily for 16 weeks	Clinical response by week 16; translational endpoints	2b
Solimani, 2019 (Front Immunol) [10]	Compassionate-use case series	Recalcitrant mucosal and/or cutaneous LP	5	Secukinumab (n = 3), Ustekinumab (n = 1), Guselkumab (n = 1)	Clinical improvement (investigator-assessed)	4
Asarch, 2009 (JAAD) [21]	Case series + literature review (pharmacovigilance)	Patients on TNF-α antagonists developing LP/lichenoid eruptions	13 cases (2 new + 11 literature)	Exposure to infliximab or adalimumab (no therapeutic intervention for LP tested)	Occurrence of LP/lichenoid eruption; clinical characterization	4
Cheng, 2012 (Cochrane) [22]	Systematic review of RCTs/controlled trials	Erosive LP (oral, anogenital, oesophageal)	15 studies; 473 participants	Topical agents (incl. pimecrolimus, aloe vera), others vs. placebo/vehicle or active	Pain improvement; global clinical improvement; adverse events	1a

Abbreviations: OLP = oral lichen planus; CLP = cutaneous lichen planus; MLP = mucosal lichen planus; LPP = lichen planopilaris; TAC = triamcinolone acetonide; ODSS = Oral Disease Severity Score; IGA = Investigator’s Global Assessment; PDT = photodynamic therapy; LLLT = low-level laser therapy. Level of evidence scale (OCEBM 2011, condensed): 1a = systematic review of RCTs; 1b = individual RCT; 2b = cohort/low-quality RCT or single-arm phase II; 4 = case series.

## 3. Immunopathogenesis of Lichen Planus: Therapeutic Target

Lichen planus (LP) is a chronic, immune-mediated, mucocutaneous disease driven primarily by T-cell-mediated cytotoxicity [1]. It is histologically characterized by interface dermatitis with a band-like lymphocytic infiltrate along the dermoepidermal junction and hydropic degeneration of basal keratinocytes [3]. Although the precise etiology remains unclear, the pathogenesis of LP is increasingly understood as a result of dysregulated immune surveillance involving cytotoxic CD8+ T lymphocytes, antigen-presenting cells, pro-inflammatory cytokines, and aberrant activation of intracellular signaling pathways [15]. At the cellular level, LP is considered a prototypical interface dermatitis, where CD8+ cytotoxic T lymphocytes (CTLs) recognize aberrant or modified self-antigens presented by keratinocytes through MHC class I molecules, possibly induced by viral infections, drugs, or autoantigen exposure [15]. These CD8+ T cells release cytotoxic mediators, including perforin, granzyme B, and Fas ligand (FasL), leading to apoptosis of basal keratinocytes. In parallel, IFN-γ produced by both CD8+ and CD4+ T cells enhances MHC class I expression on keratinocytes, amplifying antigen presentation and immune-mediated damage [7]. Dendritic cells and Langerhans cells serve as pivotal antigen-presenting cells (APCs) in LP lesions. They produce IL-12, IL-23, and type I interferons, supporting the differentiation of naïve T cells into Th1 and Th17 subsets [6]. The IL-23/Th17 axis is particularly relevant in sustaining chronic inflammation: IL-23 stabilizes Th17 cells, which produce IL-17A, IL-17F, IL-22, and GM-CSF, contributing to keratinocyte activation, neutrophilic chemotaxis, and perpetuation of tissue damage. Elevated IL-6 further skews the response toward Th17 dominance by inhibiting Treg differentiation via STAT3 phosphorylation [6]. The cytokine milieu in LP is heavily Th1- and Th17-skewed [10], with abundant expression of TNF-α, IFN-γ, IL-17, and IL-23 within lesional skin and mucosa. TNF-α, a master regulator of inflammation, enhances leukocyte recruitment, endothelial activation, and survival signaling through the NF-κB pathway, which is constitutively active in LP lesional skin. This transcription factor regulates pro-inflammatory gene expression, including IL-1β, ICAM-1, and chemokines such as CXCL9/10, which recruit additional T cells [7]. Several intracellular signaling cascades downstream of cytokine receptors are involved in LP pathogenesis.

The JAK/STAT pathway is activated by cytokines such as IFN-γ (via JAK1/JAK2 → STAT1) [8,9] and IL-6 (via JAK1/JAK2 → STAT3). STAT1/STAT3 overexpression has been documented in LP tissues, suggesting transcriptional amplification of immune effector programs. JAK inhibitors (e.g., tofacitinib, baricitinib) disrupt these cascades, reducing cytokine-driven inflammation and keratinocyte apoptosis [9,20].

The NF-κB pathway, activated through TNF receptor-associated factors (TRAFs), promotes transcription of pro-survival and inflammatory genes. Although direct pharmacologic inhibitors of NF-κB are lacking in clinical dermatology, upstream blockade via anti-TNF-α antibodies (e.g., infliximab, adalimumab) may offer indirect modulation [21].

The mTOR pathway, critical for T-cell metabolism, proliferation, and differentiation, is also implicated. mTORC1 activation supports effector T cell survival and suppresses Treg activity. mTOR inhibitors such as sirolimus have shown anecdotal success in oral LP, especially in transplant recipients [13].

Additional mediators such as CXCR3 ligands (CXCL9/10/11) and ICAM-1/VCAM-1 may facilitate lymphocyte trafficking and retention within lesional tissue, adding another layer of immune complexity [7].

**Figure 1:**
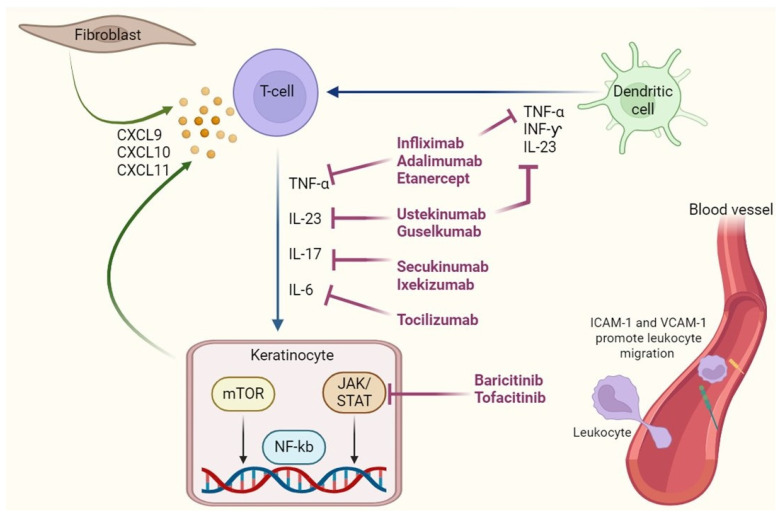
Molecular targets of immunomodulatory therapies in Lichen Planus.

From a therapeutic standpoint (Figure 1), this expanding immunopathogenic framework has allowed the identification of specific molecular targets for precision immunotherapy [16]. Currently, monoclonal antibodies directed against IL-17A (secukinumab, ixekizumab) [19], IL-23p19 (guselkumab) [10], and TNF-α (adalimumab, infliximab) [21] are in off-label or experimental use for LP. Their ability to selectively interfere with key inflammatory mediators offers a strategic advantage over broad immunosuppressants. Similarly, JAK inhibitors (e.g., tofacitinib, baricitinib) demonstrate broad efficacy by blocking signal transduction from multiple cytokines simultaneously, particularly in mucosal LP [13,14,20].

LP’s immunologic overlap with diseases like psoriasis, vitiligo, and oral lichen planus-like graft-versus-host disease further supports the relevance of shared cytokine and cellular targets, reinforcing the rationale for therapeutic cross-application [13,14,15].

Ultimately, the successful application of targeted therapies in LP will depend on deeper molecular profiling, identification of biomarkers predictive of response, and stratification of patients according to immune phenotype and disease subtype [16]. These developments are essential to shift from empirical to mechanism-based treatment approaches, optimizing efficacy while minimizing adverse effects.

Schematic representation of key cytokine pathways involved in the immunopathogenesis of lichen planus and corresponding targeted therapies. Activated dendritic cells and T cells contribute to the chronic inflammatory response through the release of pro-inflammatory cytokines, including TNF-α, IL-23, IL-17, and IL-6, which act on keratinocytes via signaling cascades such as JAK/STAT, NF-κB, and mTOR. Targeted biologics (e.g., infliximab, adalimumab, secukinumab, tocilizumab) inhibit specific cytokines, while small-molecule inhibitors (baricitinib, tofacitinib) block downstream intracellular pathways. This therapeutic blockade aims to disrupt the self-amplifying inflammatory circuit driving keratinocyte damage in lichen planus.

## 4. Overview of Traditional Therapies and Their Limitations

Timeline illustrating the progressive development of treatment options for lichen planus. Before 2000, therapy mainly relied on topical and systemic corticosteroids and broad immunosuppressive agents. In the early 2000s, off-label use of topical tacrolimus, methotrexate, and cyclosporine became more common. Since 2015, the emergence of biologic therapies targeting specific immune pathways—such as JAK/STAT (baricitinib, tofacitinib), IL-17 (secukinumab, ixekizumab), IL 12/23 (Ustekinumab), IL-23 (guselkumab), and TNF-α (infliximab, etanercept, adalimumab)—has marked a shift toward precision immunomodulation in refractory lichen planus.

Conventional treatments for lichen planus (LP) are centered on the suppression of inflammation and relief of symptoms such as pruritus, pain, and mucosal ulcerations [16]. Although many patients benefit from first-line therapies, a significant proportion—particularly those with mucosal, erosive, or generalized forms of LP—experience chronic, relapsing disease that is poorly responsive to standard approaches [22]. These clinical challenges highlight the therapeutic limitations of conventional strategies, which often rely on broadly immunosuppressive mechanisms rather than targeted immunomodulation [23].

### 4.1. Topical and Systemic Corticosteroids

Topical corticosteroids are widely considered the first-line treatment for localized LP, both cutaneous and mucosal [12]. High-potency agents such as clobetasol propionate and fluocinonide are typically applied in ointment or gel formulations, and in mouthwash suspensions for oral LP [11,12]. These agents reduce local immune cell infiltration and cytokine production, thereby alleviating erythema, erosion, and pruritus [12]. However, long-term use, especially on delicate mucosal surfaces, may lead to skin atrophy, secondary infections such as candidiasis, and delayed epithelial healing [16].

In more extensive or refractory cases, systemic corticosteroids (e.g., prednisone) are frequently employed [12]. Short-term courses may induce rapid symptom control, but chronic use is limited by well-documented adverse effects, including hyperglycemia, hypertension, osteoporosis, mood alterations, and heightened infection risk [5]. Moreover, relapses upon tapering are common, reinforcing the need for maintenance alternatives.

### 4.2. Systemic Immunosuppressive Agents

In patients who fail corticosteroid therapy or present with contraindications, several off-label systemic immunosuppressants are used, though none are approved specifically for LP.

- Cyclosporine, a calcineurin inhibitor, suppresses IL-2–mediated T-cell activation. It has shown efficacy in oral and genital LP, particularly in erosive forms. However, its clinical use is hindered by significant toxicity, including nephrotoxicity, hypertension, gingival hyperplasia, and complex pharmacokinetic interactions [5]. Topical cyclosporine has also been used for oral LP, with inconsistent results [11].
- Methotrexate, a folate pathway antagonist with both anti-proliferative and anti-inflammatory properties, is used in generalized and erosive LP [24]. While effective in selected cases, methotrexate carries risks of hepatotoxicity, bone marrow suppression, and gastrointestinal adverse effects, requiring regular laboratory monitoring [24].
- Azathioprine, a purine analog that interferes with DNA synthesis in proliferating immune cells, has been utilized in erosive oral LP and LP associated with autoimmune overlap syndromes [16,23]. Its use is complicated by variable metabolism (requiring TPMT genotyping) and risks such as myelosuppression, gastrointestinal toxicity, and infection susceptibility.
- Acitretin, a systemic retinoid and derivative of vitamin A, has shown utility particularly in hypertrophic and cutaneous LP [18]. It promotes keratinocyte differentiation and modulates epidermal proliferation while exerting mild immunomodulatory effects. Acitretin may be preferred in patients where immunosuppression is contraindicated (e.g., history of infection or malignancy) [18]. However, it is teratogenic, causes mucocutaneous dryness, hyperlipidemia, and requires strict contraceptive measures in women of childbearing potential [15].

Other immunosuppressive options include mycophenolate mofetil and hydroxychloroquine, although clinical evidence supporting their efficacy is limited to small case series and anecdotal reports. These agents are often reserved for refractory cases or when standard options are not tolerated [13].

### 4.3. Limitations and Unmet Needs

Despite a variety of available immunosuppressive agents, treatment of refractory LP—particularly oral and genital variants—remains problematic [13,15]. Many patients achieve only partial or temporary remission, while others exhibit primary non-response. Furthermore, the use of traditional systemic therapies is often constrained by significant toxicity, need for continuous laboratory surveillance, and poor suitability for long-term maintenance in a chronic condition [13].

Importantly, conventional therapies exert non-specific immunosuppressive effects, dampening overall immune activity without selectively targeting the immune pathways central to LP pathogenesis (such as the IL-17, IFN-γ, or JAK/STAT axis) [13,14]. This broad mechanism not only limits efficacy in certain disease phenotypes but also increases the risk of systemic side effects, including infection and drug-induced complications.

These limitations underscore the urgent need for novel therapeutic approaches that offer selective immune modulation, better tolerability, and durable disease control. In recent years, biologic therapies and small-molecule inhibitors have emerged as promising alternatives, guided by advances in molecular immunology and offering a more pathogenesis-driven strategy to managing LP [13,14,16].

## 5. Biologic Therapies in Lichen Planus

**Figure 2:**
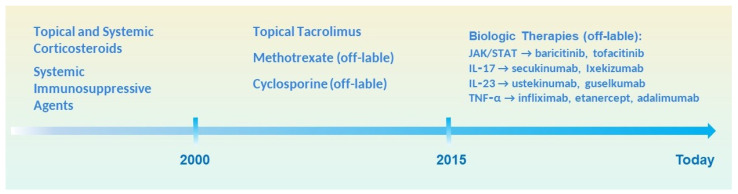
Evolution of therapeutic strategies for Lichen Planus from conventional to targeted therapies.

The treatment of lichen planus (LP) has evolved considerably with the advent of biologic agents and small-molecule inhibitors (Figure 2). These therapies have ushered in a new era of precision immunomodulation [14], shifting away from traditional broad-spectrum immunosuppression toward targeted blockade of specific cytokines, immune cell receptors, and intracellular signaling pathways [16]. This transition is supported by a deeper understanding of the immunopathogenesis of LP, particularly the roles of T-cell activation, pro-inflammatory cytokines, and the JAK/STAT and IL-23/Th17 pathways. Although randomized controlled trials are lacking, growing clinical experience, especially in treatment-refractory cases, is beginning to define the potential therapeutic niche for these advanced agents [15].

### 5.1. Anti-TNF-α Agents: Infliximab, Adalimumab, Etanercept

Tumor necrosis factor-alpha (TNF-α) is a central mediator in many chronic inflammatory diseases, including LP. It promotes keratinocyte apoptosis, endothelial activation, and leukocyte recruitment via NF-κB and MAPK signaling [21].

Infliximab, a chimeric IgG1 monoclonal antibody that binds soluble and transmembrane TNF-α, has been reported to induce remission in patients with erosive oral LP unresponsive to steroids and immunosuppressants. Infliximab acts by neutralizing TNF-α and blocking its interaction with TNFR1 and TNFR2. Some cases have shown sustained improvement; however, concerns persist regarding infusion reactions, immunogenicity, and paradoxical lichenoid eruptions [21].

Adalimumab, a fully human anti-TNF-α monoclonal antibody, has demonstrated beneficial effects in cutaneous and oral LP, including in patients with coexisting autoimmune conditions such as Crohn’s disease. Its subcutaneous administration and favorable safety profile make it a practical option in selected cases [5].

Etanercept, a fusion protein of TNFR2 and the Fc portion of IgG1, acts as a decoy receptor for TNF-α. While initial reports suggested efficacy, subsequent studies have shown inconsistent outcomes, with some patients experiencing relapse upon discontinuation [24].

Anti-TNF therapies, although potentially effective, are associated with serious adverse effects including increased susceptibility to infections, latent tuberculosis reactivation, and paradoxical induction or exacerbation of LP-like eruptions [21], especially in predisposed individuals [16].

### 5.2. Anti-IL-17 and Anti-IL-23 Agents: Secukinumab, Ixekizumab, Guselkumab (Table 2)

The IL-23/Th17 axis plays a critical role in LP by supporting the differentiation and maintenance of Th17 cells, which secrete IL-17A, IL-17F, and IL-22—cytokines that promote epithelial barrier disruption and neutrophil recruitment [10].

Secukinumab is a fully human monoclonal antibody targeting IL-17A. Case series and observational data indicate improvement in both cutaneous and mucosal LP, including resolution of erosions and reduced pain. Mechanistically, secukinumab inhibits IL-17A-mediated neutrophil chemotaxis, keratinocyte hyperproliferation, and inflammation [19].

Ixekizumab, another IL-17A inhibitor with a higher binding affinity than secukinumab, has also been used successfully in generalized LP and erosive oral LP. Its rapid onset of action and mucosal efficacy are particularly advantageous [10].

Guselkumab targets the p19 subunit of IL-23, thereby indirectly reducing IL-17 production. Though data in LP are limited, early reports in patients with erosive oral LP have demonstrated symptomatic relief and lesion regression [10,25].

Inhibitors of IL-17 and IL-23 offer the advantage of targeted immune modulation with a lower risk of global immunosuppression. They may be particularly effective in Th17-dominant phenotypes of LP [14].

**Table 2:** Overview of Monoclonal Antibodies and Small-Molecule Therapies for Lichen Planus.

Drug Name	Type	Target	Indication in LP	Comments
Infliximab	Monoclonal Antibody	TNF-α	Refractory oral LP	Infusion reactions, paradoxical LP
Adalimumab	Monoclonal Antibody	TNF-α	Cutaneous and oral LP	Subcutaneous use, favorable safety
Etanercept	Fusion Protein	TNF-α	Variable efficacy	Relapses post-treatment
Secukinumab	Monoclonal Antibody	IL-17A	Cutaneous and mucosal LP	Effective in neutrophilic inflammation
Ixekizumab	Monoclonal Antibody	IL-17A	Generalized and erosive oral LP	High binding affinity, rapid action
Guselkumab	Monoclonal Antibody	IL-23p19	Erosive oral LP	Reduces IL-17 indirectly
Tocilizumab	Monoclonal Antibody	IL-6 receptor	Anecdotal use in erosive forms	Pleiotropic cytokine role
Anakinra	Recombinant Protein	IL-1 receptor	Limited data; theoretical use	Limited clinical evidence
Tofacitinib	Small Molecule (JAK Inhibitor)	JAK1/3	Refractory oral/genital LP	Broad cytokine suppression
Baricitinib	Small Molecule (JAK Inhibitor)	JAK1/2	Exploratory use in inflammatory LP	Inhibits Th1/Th17 axis

### 5.3. Anti-IL-6 and IL-1 Inhibitors: Tocilizumab, Anakinra

IL-6 is a pleiotropic cytokine that contributes to Th17 polarization and JAK/STAT3 activation [26]. Tocilizumab, an IL-6 receptor-blocking monoclonal antibody, is approved for rheumatoid arthritis and has been tested in LP with anecdotal reports of efficacy, especially in erosive forms. However, robust evidence is lacking [27,28].

Anakinra, a recombinant IL-1 receptor antagonist, blocks IL-1α and IL-1β signaling. IL-1 is an upstream mediator in several autoinflammatory cascades [29]. Although theoretical rationale exists for its use in LP, particularly in syndromic or overlap presentations, published data remain minimal [30]. These agents may have future value in LP subsets with a strong autoinflammatory component [31].

### 5.4. JAK Inhibitors and Targeted Small Molecules: Tofacitinib, Baricitinib (Table 2)

JAK inhibitors block intracellular signal transduction downstream of multiple cytokine receptors, including those for IFN-γ, IL-6, and IL-12/23 [32]. This makes them uniquely suited to diseases like LP with multifaceted cytokine dysregulation [33,34].

Tofacitinib, a JAK1/3 inhibitor, has been shown to induce clinical remission in several cases of refractory oral and genital LP, often within 2 to 6 weeks. Its broad cytokine suppression includes IL-2, IL-6, IFN-γ, and IL-15, which may underlie its efficacy in reducing T-cell activity and keratinocyte apoptosis [35].

Baricitinib, a selective JAK1/2 inhibitor, has also demonstrated benefit in inflammatory dermatoses and is being explored off-label for LP [20]. Its inhibition of STAT1/3 phosphorylation may dampen the Th1 and Th17 pathways simultaneously [20].

The oral administration, rapid onset, and broad immunoregulatory profile of JAK inhibitors make them promising agents, particularly for erosive mucosal LP and in patients intolerant to biologics. However, concerns remain regarding herpes zoster reactivation, lipid dysregulation, and long-term safety [36].

### 5.5. Emerging Targets: PD-1/PD-L1, BTK Inhibitors, Anti-IFN-γ

Expanding knowledge of LP immunobiology has revealed additional therapeutic targets [37].

The PD-1/PD-L1 axis plays a critical role in maintaining peripheral immune tolerance by inhibiting autoreactive T cell responses. In lichen planus (LP), multiple studies have demonstrated that expression of PD-1 and PD-L1 is significantly reduced in lesional and nonlesional LP skin compared to healthy controls, suggesting that impaired PD-1/PD-L1 signaling may contribute to the loss of tolerance and the persistence of cytotoxic T cell-mediated inflammation characteristic of LP. This is supported by immunohistochemical and ELISA-based studies showing lower PD-1/PD-L1 levels in LP tissue, and by the observation that decreased checkpoint signaling may facilitate the dense lymphocytic infiltrate targeting basal keratinocytes [38,39].

Clinically, immune checkpoint blockade with anti-PD-1/PD-L1 agents such as nivolumab and pembrolizumab frequently induces lichenoid eruptions, including lichen planus and lichen planus pemphigoides, as a form of immune-related adverse event. These eruptions are histologically and clinically indistinguishable from idiopathic LP, and their development is attributed to the removal of PD-1–mediated inhibition, which unleashes autoreactive T cells against cutaneous and mucosal antigens. Experimental models further confirm that PD-1 is essential for restraining effector CD8+ T cell activity in the skin, and its absence leads to local tissue pathology resembling LP [40,41,42,43,44].

Enhancing PD-1 signaling is theoretically attractive as a means to dampen pathogenic T cell responses in LP, but this approach remains experimental and has not been tested in clinical trials. Current evidence supports the concept that defective PD-1/PD-L1 signaling is a permissive factor in LP pathogenesis, and that immune checkpoint blockade can unmask or exacerbate lichenoid autoimmunity [38,39,41,42,44,45]. While the PD-1/PD-L1 axis is mechanistically relevant, current evidence in LP is indirect, largely derived from checkpoint-inhibitor–associated lichenoid eruptions rather than interventional studies treating LP itself.

BTK is a critical signaling molecule in B cells, as well as in myeloid cells, mast cells, and other hematopoietic lineages. Inhibition of BTK disrupts B cell receptor (BCR) signaling, impairs autoantibody production, and modulates Fc receptor and Toll-like receptor pathways, thereby attenuating both humoral and innate immune responses [46,47,48]. In autoimmune skin diseases with pathogenic autoantibodies (e.g., pemphigus), BTK inhibition has shown promise by reducing B cell activation and autoantibody-mediated tissue injury [48,49]. However, LP is primarily a T cell–mediated interface dermatitis, and the pathogenic role of B cells or autoantibodies in classic LP is not established [50].

BTK inhibition could theoretically impact LP pathogenesis in rare or overlap subtypes with significant humoral or antigen-driven components, but this remains speculative. The safety profile of first-generation BTK inhibitors (e.g., ibrutinib) includes dermatologic adverse events and off-target effects, while next-generation agents are being developed to improve tolerability. While BTK inhibitors have demonstrated efficacy in B cell malignancies and are under investigation for several autoimmune and immune-mediated dermatological diseases (e.g., pemphigus, chronic spontaneous urticaria, systemic lupus erythematosus), no published clinical trials or case series have evaluated their use in LP [49,50,51,52]. Bruton’s tyrosine kinase (BTK) inhibitors are discussed as investigational based on immunologic plausibility; to date, there are no prospective trials or clinically meaningful case series in LP, and any potential role remains theoretical. Given the absence of LP-specific clinical outcomes, BTK inhibition should be considered a research priority rather than a near-term therapeutic option.

### 5.6. IL-4/IL-13 Inhibition: The Case of Dupilumab

Dupilumab, a fully human monoclonal antibody targeting the interleukin-4 receptor alpha (IL-4Rα), blocks both IL-4 and IL-13 signaling and is approved for several type 2 inflammatory conditions, including atopic dermatitis, asthma, and chronic rhinosinusitis with nasal polyposis. Its mechanism of action primarily affects Th2-mediated immune responses, which are not predominant in the immunopathogenesis of lichen planus (LP), characterized by elevated IFN-γ, IL-17, and IL-23 expression [6].

Evidence for IL-4Rα blockade in LP is limited to isolated case reports and small series with mixed outcomes, without randomized or controlled data supporting efficacy.

Systematic reviews of off-label use in inflammatory skin diseases do not recognize LP as a validated indication, and the immunopathogenic mismatch raises concerns about its theoretical suitability.

Nonetheless, emerging evidence suggests that dupilumab may offer symptomatic relief from pruritus, one of the most burdensome symptoms in LP [53,54]. Pilot real-life studies in adolescents with atopic dermatitis have demonstrated a rapid and sustained reduction in pruritus following dupilumab administration, suggesting a potential mechanistic benefit even beyond strict Th2-driven contexts [54]. This antipruritic effect may be mediated through suppression of sensory neuron-IL-4R signaling, which plays a role in chronic itch independent of lesion type [53].

However, caution is warranted. Paradoxical lichenoid reactions, including new-onset lichen planus-like eruptions during dupilumab therapy, have been reported. A documented case by Mastorino et al. describes the development of lichen ruber planus during treatment for atopic dermatitis, raising the possibility that IL-4/IL-13 blockade may disrupt immune homeostasis in susceptible individuals, potentially unmasking or triggering LP via skewing toward Th1/Th17 dominance [55].

Paradoxical LP-like eruptions during dupilumab therapy have been reported; taken together, IL-4Rα blockade should not be considered an established treatment for LP and, if used off-label, warrants careful risk–benefit discussion and monitoring. However, its potential utility in select cases with severe pruritus, or in patients with overlapping atopic diathesis, merits further investigation. Future studies should explore whether specific LP subtypes or pruritus-dominant phenotypes might benefit from targeted IL-4/IL-13 blockade, ideally within prospective, biomarker-stratified clinical trials.

## 6. Research Gaps and Future Directions

Despite encouraging clinical signals from case reports and observational data, the use of biologic and small-molecule therapies in lichen planus (LP) remains largely exploratory [6,14]. Several important gaps in the current evidence base must be addressed to enable broader clinical adoption and regulatory validation of these treatments [16].

### 6.1. Lack of High-Quality Randomized Controlled Trials

One of the most pressing limitations is the absence of randomized controlled trials (RCTs) evaluating biologics and JAK inhibitors in LP [14]. Existing data are dominated by small-scale, uncontrolled case series and isolated reports. Without rigorously designed RCTs—including appropriate blinding, control arms, and standardized endpoints—it is difficult to draw robust conclusions about efficacy, safety, and comparative effectiveness across agents. Regulatory approval and inclusion in clinical guidelines will require high-level evidence from phase II/III studies.

### 6.2. Phenotypic Heterogeneity and Stratification Needs

LP is a clinically and histologically heterogeneous disease, encompassing various subtypes such as cutaneous, oral, genital, nail, and follicular forms [6]. Notably, therapeutic response and disease burden vary substantially across LP subtypes. Oral LP often exhibits chronic and erosive behavior requiring long-term immunosuppression, whereas cutaneous LP may resolve spontaneously or respond to short courses of therapy. Genital and hypertrophic variants frequently prove refractory and significantly impair quality of life. These differences impact both treatment selection and the interpretation of efficacy data. Moreover, the inconsistent use of outcome measures across studies—ranging from non-standardized clinical scores to subjective symptom scales (Table 1)—further limits comparability and synthesis. As a result, caution is warranted when extrapolating data from one LP subtype to another, especially in trials enrolling mixed or poorly characterized populations [37]. Future research must address this heterogeneity by defining clear diagnostic criteria and developing subtype-specific outcome measures [16]. Stratifying patients by clinical phenotype, immune profile, and comorbidity burden will enable more targeted and meaningful assessments of therapeutic efficacy.

### 6.3. Need for Personalized and Precision Medicine Approaches

As the understanding of LP immunopathogenesis evolves, so too does the opportunity for a precision medicine approach [7]. Identifying biomarkers predictive of treatment response—such as cytokine signatures, gene expression profiles, or immunophenotyping—could guide individualized therapy selection. For example, IL-17 inhibitors may be more effective in Th17-dominant disease, while JAK inhibitors may benefit patients with broader cytokine dysregulation [10]. Integrating immunologic profiling into clinical trials will be crucial to defining responder subsets and minimizing trial-and-error prescribing [7,16].

### 6.4. Proposals for Future Studies

To advance the field, future research should prioritize:

- Multicenter, randomized controlled trials assessing specific agents across LP subtypes
- Development and validation of LP-specific clinical scores and patient-reported outcome measures (PROMs)
- Longitudinal registries to monitor real-world effectiveness and long-term safety
- Integration of biomarker discovery and immune profiling into clinical study design
- Exploration of combination therapies, such as biologics with low-dose systemic immunomodulators or topical agents

Finally, collaborative efforts among dermatologists, oral medicine specialists, immunologists, and industry partners will be critical to overcoming current challenges [5]. Establishing standardized treatment pathways and consensus guidelines will help translate emerging therapies into routine clinical practice and improve outcomes for patients with this chronic and often debilitating disease [14].

## 7. Discussion

Lichen planus (LP) is a chronic immune-mediated disease involving autoreactive CD8+ T cells, pro-inflammatory cytokines, and antigen-presenting cells, leading to basal keratinocyte apoptosis and mucocutaneous inflammation. Although LP is relatively common and burdensome—particularly in its oral, genital, and hypertrophic forms—therapeutic options remain limited. Traditional immunosuppressants (e.g., corticosteroids, calcineurin inhibitors, methotrexate) often lack sustained efficacy and specificity, with significant side effects.

Recent advances have clarified key pathogenic pathways, particularly the Th1/IFN-γ and IL-23/Th17/IL-17 axes, which sustain inflammation and epithelial damage. These insights have enabled the development of targeted therapies. Biologic agents such as IL-17 inhibitors (secukinumab, ixekizumab) and IL-23p19 blockers (guselkumab) have demonstrated promising results, particularly in mucosal LP. We have clarified the distinction between dual IL-12/23 blockade (ustekinumab) and selective IL-23 inhibition, given their mechanistic and clinical differences.

JAK inhibitors (e.g., tofacitinib, baricitinib) also show potential by broadly suppressing cytokine networks implicated in LP, especially in refractory cases. Their oral route and rapid onset make them attractive options, though long-term safety remains to be established.

Other investigational strategies—such as BTK inhibitors, PD-1 modulation, IFN-γ blockade, and dupilumab—are currently speculative, with limited or indirect evidence. We have revised the manuscript to emphasize the preliminary nature of these approaches and avoid overstating their clinical readiness.

Despite encouraging developments, most data stem from small series or case reports. The marked clinical heterogeneity of LP subtypes and the lack of validated biomarkers continue to hinder treatment standardization and precision care.

## 8. Conclusions

LP exemplifies the evolving interface between dermatology and immunology, where molecular insights increasingly inform therapeutic decisions. Biologic agents and small-molecule inhibitors represent a promising leap forward, offering improved efficacy and safety in difficult-to-treat cases. As research progresses, the integration of immunopathologic data, biomarker stratification, and individualized therapeutic algorithms will be essential to fully realize the potential of precision medicine in LP. Through collaborative clinical trials and translational studies, the goal of durable, targeted, and well-tolerated remission is becoming increasingly attainable.

## Data Availability

The data presented in this study were obtained from public domain resources such as PubMed, Scopus, and Web of Science.
